# How I do it: microsurgical extraforaminal sequestrectomy through a midline intertransverse approach

**DOI:** 10.1007/s00701-026-07040-w

**Published:** 2026-09-23

**Authors:** Victor E. Staartjes, Adrian Elmi-Terander, Gunnar Nilsson, Mario Ruiz

**Affiliations:** 1 Department of Clinical Neuroscience, Karolinska Institutet, Stockholm, Sweden; 2 Capio Spine Center Stockholm, Löwenströmska Hospital, Upplands Väsby, Sweden; 3 Machine Intelligence in Clinical Neuroscience & Microsurgical Neuroanatomy (MICN) Laboratory, Department of Neurosurgery, Clinical Neuroscience Center, University Hospital Zurich, University of Zurich, Rämistrasse 100, CH-8091 Zurich, Switzerland; 4 Department of Surgical Sciences, Uppsala University, Uppsala, Sweden; 5 Department of Medical Sciences, Örebro University, Örebro, Sweden

**Keywords:** Extraforaminal disc herniation, Far lateral disc herniation, Intertransverse approach, Lumbar microdiscectomy, Sequestrectomy, Surgical video

## Abstract

**Background:**

Several surgical approaches exist for extraforaminal (far lateral) lumbar disc herniations, including paraspinal Wiltse-type, interlaminar, lateral intertransverse, and combined medial–lateral techniques.

**Method:**

We present microsurgical extraforaminal sequestrectomy through a midline intertransverse approach, chosen for familiar anatomy, minimal facet joint resection, and avoidance of a muscle-splitting trajectory. Unilateral subperiosteal dissection exposes the isthmus, facet margin, and transverse processes with the intertransverse ligament; the facet margin is removed with a straight osteotome, the ligament resected, the intertransverse artery coagulated, and the nerve root and disc space exposed for sequestrectomy.

**Conclusion:**

This approach provides effective sequestrectomy while preserving spinal stability using familiar midline anatomy.

**Supplementary Information:**

The online version contains supplementary material available at https://doi.org/10.1007/s00701-026-07040-w.

## Relevant surgical anatomy

**Fig. 1:**
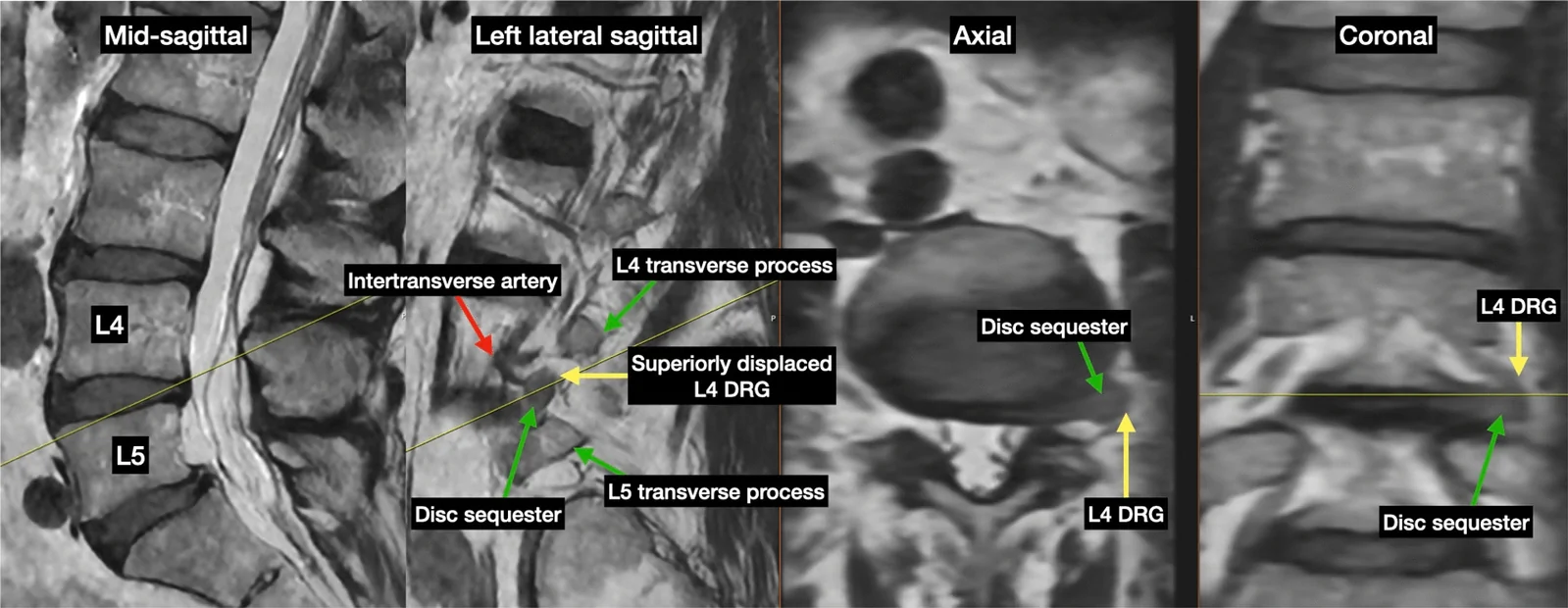
T2-weighted MR imaging showing a left-sided extraforaminal disc herniation at L4-L5 that lifts the dorsal root ganglion of the L4 nerve upwards, with coronal, axial, left lateral sagittal, and mid-sagittal views. Note the close relationship of the disc sequester and superiorly displaced L4 dorsal root ganglion with the intertransverse artery, arising from the lumbar segmental artery

**Fig. 2:**
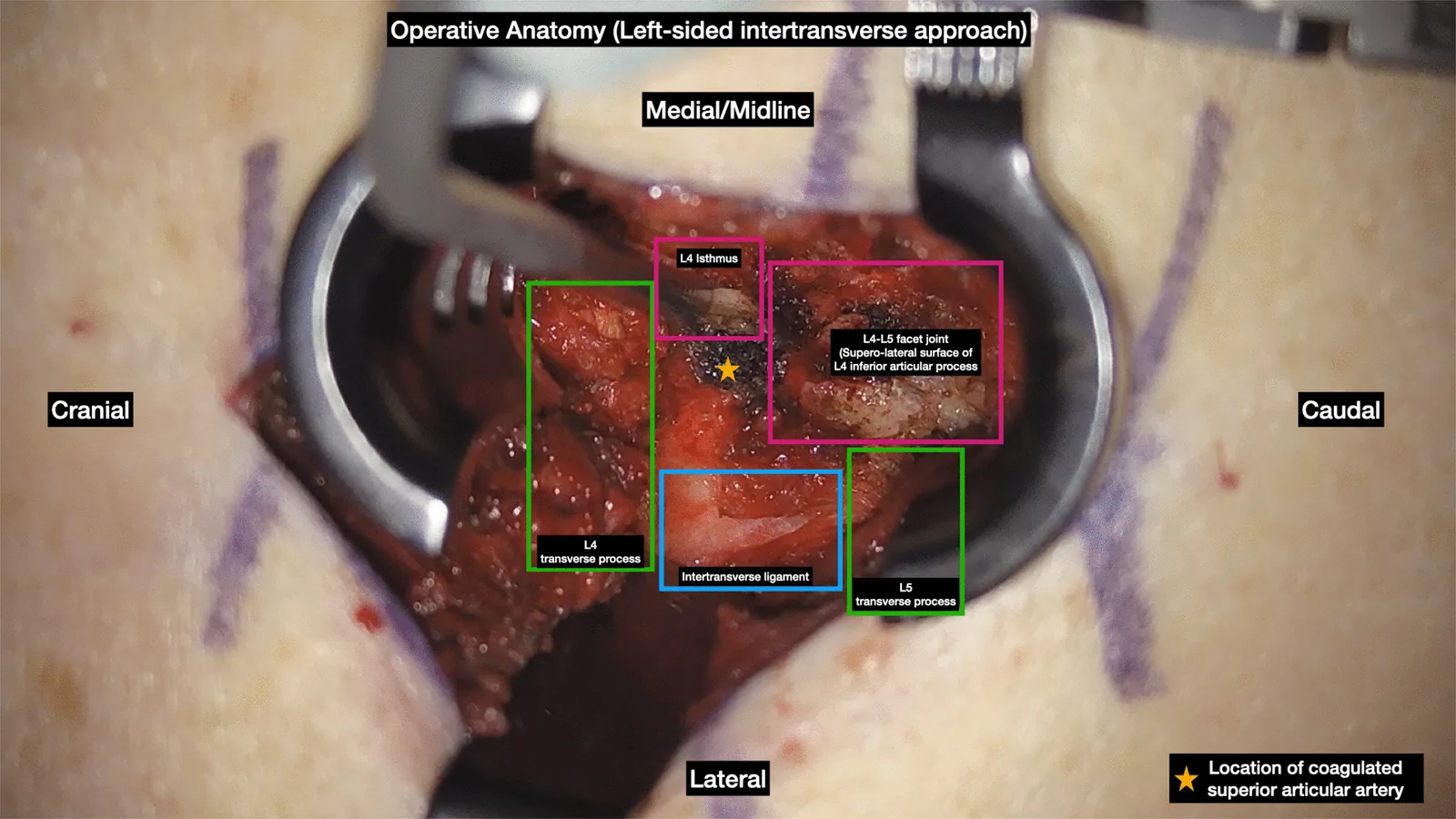
Operative anatomy of the left-sided intertransverse approach, showing the L4 isthmus, the L4-L5 facet joint (supero-lateral surface of the L4 inferior articular process), the L4 and L5 transverse processes, the intertransverse ligament, and the location of the coagulated superior articular artery

On MR imaging, the disc sequester and the superiorly displaced L4 dorsal root ganglion lie in close relationship to the intertransverse artery, which arises from the lumbar segmental artery (Fig. 1). The operative anatomy of the midline intertransverse approach comprises the L4 isthmus, the supero-lateral surface of the L4-L5 facet joint (the L4 inferior articular process), the L4 and L5 transverse processes, and the intertransverse ligament, with the superior articular (intertransverse) artery piercing the ligament (Fig. 2).

## Description of the technique

We illustrate this technique in a 72-year-old man with sudden electric shock-like radiculopathy from the left buttock into the shin and big toe, without improvement after 7 weeks. Clinical examination showed M3 weakness of the foot and big toe extensors on the left and hypesthesia over the medial foot edge. MR imaging revealed a left-sided extraforaminal disc herniation at L4-L5 that lifts the dorsal root ganglion (DRG) of the L4 nerve upwards (Fig. 1). A diagnostic nerve root block at L4 left with 10 mg lidocaine and 40 mg methylprednisolone led to significant pain improvement with persistent numbness.

The patient is positioned prone on a Wilson frame, which is slightly kyphosed. Sterile prepping and draping is followed by fluoroscopic level verification using a needle. Subcutaneous and unilateral intramuscular infiltration with ropivacaine is performed, and antibiotics are administered (2 g i.v. cloxacillin). A midline skin incision (5 cm length) is made, centered slightly above the index level. Monopolar preparation is carried through the subcutaneous fat, with unilateral opening of the fascia and subperiosteal dissection, exposing the isthmus of the superior level (L4), the index level's facet joint's supero-lateral margin (L4-L5), and the L4 and L5 transverse processes and connecting intertransverse ligament (Fig. 2). A Caspar retractor is placed and the index level is fluoroscopically verified.

The supero-lateral margin of the L4-L5 facet joint (L4 inferior articular process and L5 superior articular process) is removed using a 4 mm straight osteotome. The lateral isthmus is thinned and the intertransverse ligament removed using a 3 mm Kerrison rongeur, exposing the perineural fat. The intertransverse/superior articular artery, which pierces the intertransverse ligament, is coagulated with bipolar cautery.

Blunt dissection of the perineural fat exposes the L4 nerve root/DRG and disc space. Sequestrectomy is performed, the space is flushed with saline to identify any loose sequesters, and it is verified that the L4 DRG now runs freely. 40 mg of methylprednisolone is instilled along the nerve root. Closure: fascia with Vicryl 1, subcutis with Vicryl 2–0, and cutis with Monocryl 3–0, with intramuscular ropivacaine infiltration.

Postoperatively, blood loss was 30 mL. Two postoperative doses of 2 g cloxacillin (one i.v., one oral) were administered; no antithrombotic prophylaxis was given. No mobilization restrictions were set. The patient was discharged home the same day; no follow-up or postoperative imaging was planned. The patient reported no more radiating leg pain and was able to return to activities of daily living within a few days.

## Indications

The clinical entity of “far lateral”/extraforaminal disc herniation was first described by Abdullah et al. in 1974 [1], and represents only a few percent (7–12%) of herniations, most common at L4-L5 [2]. Clinical presentation is typically dominated by severe, often excruciating radicular pain from direct dorsal root ganglion compression within the narrow extraforaminal corridor [2]. Conservative management is less successful (around 10%) than for recessal disc herniations [2].

Several surgical approaches to extraforaminal disc herniations exist: paraspinal approaches, including the open Wiltse approach [9, 10] and the tubular/endoscopic Wiltse approach [3]; and midline approaches, including the intertransverse (lateral) approach [5, 7, 8], interlaminar (medial) approaches with or without facetectomy or with pars removal [6], and combined medial/lateral approaches [4]. We chose the midline intertransverse approach for its familiar midline anatomy, minimal facet joint resection necessary, avoidance of a muscle-splitting approach, and usefulness when combined intra-extraspinal pathology is present.

## Limitations

The main trade-off of the midline intertransverse approach is exposure: because it relies on a familiar midline trajectory rather than a paraspinal or tubular corridor, it requires a comparatively larger skin incision (5 cm) and more extensive subperiosteal dissection to reach the transverse processes and intertransverse ligament. Muscle-splitting paraspinal and endoscopic/tubular techniques achieve a smaller, more targeted exposure of the extraforaminal space, but only with a certain degree of routine and dedicated equipment; the midline approach trades some of that minimal invasiveness for anatomy that most spine surgeons already navigate routinely.

## How to avoid complications

Two structures deserve particular attention. The intertransverse (superior articular) artery runs directly across the operative corridor and pierces the intertransverse ligament; because it lies deep within a narrow exposure, it should be deliberately identified and coagulated with bipolar cautery as the ligament is taken down, rather than encountered unexpectedly. Because the trajectory is more lateral than a standard interlaminar approach, wrong-level surgery is also a realistic risk, so the index level should be verified fluoroscopically both before incision and again once the transverse processes and facet joint are exposed, before any bone or ligament is removed. Finally, the DRG is inevitably manipulated during exposure and sequestrectomy, which accounts for the transient dysesthesias reported in around 20% of patients postoperatively; keeping this manipulation as gentle and limited as possible is the main way to reduce their occurrence.

## Specific information for the patient

Surgical decompression by sequestrectomy yields favorable results in roughly 70–90% of patients, independent of approach [2, 4, 6]. Transient dysesthesias are sometimes (around 20%) reported postoperatively due to manipulation of the DRG.

## Supplementary Information

Below is the link to the electronic supplementary material.

Supplementary file1 Operative video demonstrating microsurgical extraforaminal sequestrectomy via a midline intertransverse approach (MP4 445590 KB)

## Data Availability

No datasets were generated or analysed during the current study.
